# Teacher-centric educational recommender systems in K12 practice: Usage and evaluation

**DOI:** 10.1016/j.heliyon.2025.e42012

**Published:** 2025-01-16

**Authors:** Sohum M. Bhatt, Katrien Verbert, Wim Van Den Noortgate

**Affiliations:** a Faculty of Psychology and Educational Sciences and imec research group itec, KU Leuven, Belgium; b Human and Computer Interaction Group, Department of Computer Science, KU Leuven, Belgium

**Keywords:** Data science applications in education, Elementary education, Improving classroom teaching, Secondary education

## Abstract

Personalized learning is hard to apply in classroom practice due to the workload of teachers. Recommender systems can help teachers make choices and hence personalize the learning environment. However, few studies have investigated how teachers use recommender systems in their practice. Educational recommender systems also require more investigation into how teachers assess recommendations to provide better systems in the future. Therefore, this research aimed to provide a better insight into the practical application of a teacher-centric educational recommender system, conduct a user-centric evaluation of the recommender system, and present teacher beliefs on the use of educational recommender systems for personalized learning. Our results indicated that teachers similarly used the recommender system by creating new lessons rather than adapting suggested lessons. Their evaluations of the recommender system highlighted the importance of accuracy and utility. Diversity and serendipity were considered to be less important. Teachers perceived recommender systems as a first step to personalized learning and as a tool for simplifying such learning. However, the current system did not affect the quality of personalized learning. Based on these results, we propose to focus more on accuracy and utility as opposed to diversity and serendipity for teacher-centric recommender system evaluation. For personalized learning, we conclude that, according to teachers, recommender systems can lead to more effective personalization by allowing teachers to focus on student needs.

## Highlights

• We present a model of teacher recommender system use.
• Common user-centric evaluation poorly evaluates teacher-centric recommender systems.
• Teachers say recommendations should match subject area and student level.
• Teacher-centric recommender systems are believed to reduce teacher workload.

## 1 Introduction

Educational recommender systems (ERSs) have been increasingly used in various contexts, such as university or online, postgraduate contexts. Recommender systems are tools that filter information according to personal relevance to each user [1]. In the context of formal primary and secondary school environment (hereafter referred to as K12), ERSs mainly recommend exercises for practice or personalized learning. The majority of ERSs provide recommendations to students (student-centric), with few of them providing recommendations for teachers (teacher-centric). Nevertheless, K12 ERSs help reduce workload by alleviating the need for constant monitoring of student abilities, enabling the personalization of teacher-specific options in the classrooms based on students’ work. The focus of most recommender systems on students is also reflected in research: there are few studies on teacher-centric ERSs in K12. More importantly, there is a dearth of studies that investigated why recommendations are used and how teachers integrate recommendations into their teaching practice [2].

For personalized learning, many school systems conceptualize personalized learning as learning with a teacher in a classroom aided by digital tools, which is referred to as digital personalized learning (DPL). In K12 DPL, teachers promote differentiation within the classroom in combination with digital tools. These digital tools typically adapt to a student's strengths and weaknesses, so do student-centric ERSs. However, despite the use of these digital tools, the implementation of personalized learning in K12 classrooms remains difficult. For teachers, DPL results in increased workload due to the need to monitor and alter learning experiences for students [3]. Fortunately, teachers also note that different digital tools can help alleviate workload [4]. We herein propose the use of teacher-centric ERSs that can help find appropriate activities and materials for their classroom, personalized to the choices of students, grade level, or other salient contexts [5]. However, there is a gap in research investigating personalized learning with teacher-centric ERSs.

In addition, there are few ERS evaluations. Some of these evaluations focused on students as users of recommender systems, assessing their use in terms of usability metrics. When educational measures are included, recommender systems are evaluated on how they improve student scores. Moreover, their use is often evaluated through system metrics rather than observation or discussion with teachers [2]. Therefore, it is unknown how teachers view and choose recommendations to use in their classrooms.

Furthermore, there is a lack of understanding on the use of teacher-centric ERSs in the K12 context. This is further exacerbated by the use of offline, accuracy-focused evaluations of the developed K12 ERSs, often resulting in different findings from user-centric evaluations [6,7]. Moreover, studies exploring which metrics and concepts are important for teacher-centric evaluations in K12 are scarce. Therefore, our first objective is to conduct a qualitative exploration on the quality and impact of a teacher-centric ERS developed for personalized learning with teachers using the recommender system in their regular practice. We also aimed to understand how the teachers in this study use the ERS for their work and what recommendations they choose. Our second objective is to determine whether user-centric frameworks adequately capture teachers' desires and opinions during ERS evaluation. With our teacher insights, additional surveys, and interviews, we also evaluate the recommender system we developed. As a third research objective, we aim to understand how recommendations impact teachers’ work when delivering DPL.

Therefore, we strive to answer three research questions in an ecologically valid scenario in K12 schools.

1. How do teachers use an ERS developed for personalized learning in the K12 context in their usual practice and contexts?
2. What are teachers' perceptions toward the ERS used?
3. How do teachers in our sample view this ERS and other recommender systems for teachers contributing to personalized learning in K12 education?

Although one specific teacher-centric ERS is investigated in this study, the intention is to provide transferable insights that may be useful in the development and evaluation of future teacher-centric ERSs in different contexts. More specifically, the research contributions are threefold: First, we identify core characteristics for teacher-centric ERSs, including personalization based on subject and difficulty level as well as the need for control over which recommendations are presented. Second, we conduct a teacher-centric evaluation of ERS for personalized learning and explore the important and unimportant aspects of the ResQue user-centric evaluation framework in teaching contexts. The teachers in our study suggest that more importance should be given to accuracy and utility as opposed to diversity and serendipity that are often important in other contexts. Utility focuses on workload-reducing potential, whereas accuracy focuses on the congruency between the subject and the level of an item as well as the choices made in the selection stage. Finally, the teachers in our study believe that recommender systems can improve the quality of DPL by allowing them to focus more on addressing student needs and differences. This provides more evidence on how technology could be used to aid in the delivery of personalized learning, filling a gap in the personalized learning literature.

## 2 Related work

### 2.1 Personalized learning

In the past 10 years, personalized learning has grown into a major focus for researchers and practitioners [8]. While the definitions of personalized learning are divided, DPL, a subset of personalized learning that focuses on the use of personalized learning aided by digital tools, can be defined as education that uses an adaptive digital learning environment to optimize cognitive, metacognitive, or motivational outcomes [9]. DPL commonly takes place in a classroom, where teachers use DPL tools to teach students a subject supplemented with further instruction or classroom discussion [10]. Importantly, DPL is often composed of small groups, where students of similar skills work on similar materials designed by teachers either individually or together for assignments [10,11]. For example, teachers can start with a lecture on English and then use a learning tool for different assignments for groups of students based on the teacher's understanding of those students' abilities. Assignments then adapt differently for each student based on mistakes within that tool. Studies on the effects of DPL have reported benefits of stronger learning outcomes, increased self-regulatory ability in students, and greater motivation than traditional learning [11,12].

The quality of DPL, and therefore the benefits of DPL, is at least partially dependent on the teacher's knowledge and ability to differentiate within the classroom [8,13]. This is particularly true in K12 education as teachers control the majority of activity in preuniversity education [14]. It has been demonstrated that the implementation of personalized learning is difficult due to the increase in workload from the manual testing of each student and the need to regularly change learning materials for all students [3]. This increased workload is also called increased orchestration load for the greater work for the teacher in orchestrating classroom activities [15]. In general, researchers attribute the load to the necessity of the teacher to observe all students, monitor data from various assessments and other sources, and regularly alter learning experiences from data and observations [15,16].

How much responsibility is given to that educational technology is also unclear. Molenaar [17] proposed a model in which teachers and educational technology lie on a continuum of automation, with one end being fully teacher-controlled and the other end being fully automated. Within this continuum, the technology can provide supportive information (teacher assistance), control some aspects of education with teacher monitoring (partial automation), or control a great deal of the classroom experience with the teacher only given alerts or monitoring specific choices (conditional or high automation, respectively). Importantly, this model considers this hybrid human–artificial intelligence (AI) interaction as beneficial for both humans and AI to address their respective shortcomings.

### 2.2 Educational recommender systems

Recommender systems can provide personalized options to alter learning experiences without teacher involvement, thus decreasing the orchestration load of personalized learning [18]. For example, if teachers would like to include an activity in their teaching, tailored to a specific group in their classroom, a recommender system can suggest activities for that group tailored to previously completed activities and general student interests, reducing the need of the teacher to know about what activities are available. As such, recommender systems could increase the use and quality of personalized learning by allowing teachers to devote more cognitive attention toward better personalization.

There is an increasing number of studies on how to implement recommender systems for teachers [19]. For example, Zapata et al. [20] developed a hybrid recommender system that uses collaborative filtering, a method that uses user data to recommend items that have been used by similar users, and content-based recommendation, a method that uses item data to recommend items similar to those that have been used and recommend learning materials for groups of teachers in university education. Another study on teacher-centric ERSs compared various collaborative filtering and content-based models using patterns of downloading materials from open educational materials for educational stakeholders [7]. Another recent content-based approach recommends learning materials based on previously read ones [21]. This recommender system originated from a model proposed by Ref. [23]. This study suggests a circular, three-stage model of before-class, during-class, and after-class steps where educational technology can be integrated to improve the quality of individual actions within those steps. Specifically, they propose that recommender systems improve the quality of finding resources as a before-class step by integrating students’ past actions, habits, and interests to provide greater personalization. The recommender system uses latent Dirichlet allocation, a probabilistic algorithm from natural language processing, to recommend learning resources from a corpus of educational resources based on similarity to previously read materials in terms of topic. During evaluation, the use of the recommender system by students and teachers during and after lessons improved the learning outcomes and communication between teachers and students. However, unlike other teacher-centric recommender systems, this system was intended for use by both teachers and students, and teacher use was never tested. Another recent teacher-centric recommender system suggested actions to improve teaching in a university course based on the previous exam results, learning outcomes, course size, and number of topics in the course [22]. Using historical course data, the authors found that the use of a support vector machine or the k-nearest neighbors algorithm for multiclass classification produces the most accurate recommendations, though this was never tested in a real setting. Finally, Yacobson et al. [2] proposed a simpler recommender system based on social trust. In this system, teachers are recommended items that have been downloaded and used by other teachers in a social network. The utilization of this recommender system increased the usage of learning resources, a criterion for usefulness in this study. However, this conclusion only held true in communities with strong social ties.

There are only a few recommender systems for K12. Of the aforementioned ERSs, only the ERS of Yacobson et al. [5] was designed and used for the K12 context. Most ERSs were used in university or postgraduate contexts, where education is more specialized and students are more in control of their own learning [23]. Contrarily, K12 education focuses on specific subjects with guidance from governments taught by teachers with a wide variety of learner contexts, such as discipline or cultural impacts [24]. With the general rise of ERSs, there is a temptation to use student-focused recommender systems to aid teachers. However, it is likely insufficient to use student-centric ERSs for teachers as such ERSs do not consider teacher decision-making capabilities, such as deciding on the pedagogy of multiple students based on educational theory or accounting for knowledge of common errors in a subject [25,26]. In other educational technologies, such as educational dashboards, these capabilities result in different patterns of use to translate insights provided by educational technology to action [27]. These patterns of use in educational dashboards begin with the awareness stage, where teachers first find the information given to them. Next, teachers move to the interpretive stage, focusing on understanding information based on their pedagogical training and data literacy [27]. Finally, in the action stage, teachers act based on their understanding of presented data, which can be making a decision (e.g., giving more support to a particular student), or reflecting upon their own practices to make a change in the future. However, there is no such model for teacher-focused ERSs due to the novelty of the field. As such, there is a need to investigate the use of ERSs for teachers and, in particular, for K12 teachers.

In addition, regardless of the context, evaluations of ERSs are urgently needed. In a literature review of ERSs, an alarming proportion of studies does not include an evaluative aspect [28]. This is especially true for teacher-centric ERSs [19]. In particular, there are increasing calls for user-centric evaluations [2,7,29]. Such evaluations focus on user-specific criteria, such as satisfaction or utility, i.e., how useful the recommender system in question is. Erdt et al. [28] also noted that there must be a greater focus on measures concentrated on education in evaluations of ERSs, such as grades. However, educational measures are not just scores based on exams or other pedagogical evaluations. For teachers in K12 education, it is unknown what criteria (both in general and in terms of educational measures) are important in the evaluation of a recommender system. In the evaluations of teacher-centric ERSs, the most common criterion is teacher satisfaction [19]. This measure focuses on the utility of recommendations and how acceptable the results are for the teacher. However, previous research on teacher satisfaction does not necessarily correlate with behavior or utility [2]. Beyond satisfaction, one such user-centric evaluation protocol previously used in a study is the ResQue framework, an evaluative framework that focuses on criteria beyond accuracy, such as utility, novelty, and diversity of recommendations [6]. Fazeli et al. [6] tested a teacher-centric recommender system that recommends educational resources to various educational stakeholders. This study compared data-centric evaluations of accuracy with user-centric evaluations and found that the former do not agree with the latter, calling for more user-centric ERS evaluations. Furthermore, the accuracy of recommendations does not have a significant impact on how teachers choose different recommender systems. However, these were only tested with surveys and did not fully explore teacher opinions qualitatively. In fact, few teacher-centric recommender systems have been tested in real-world scenarios. In addition, it has been argued that it is necessary to understand why recommendations are explored and considered to be useful for teachers [2]. This study aimed to address these research gaps. More specifically, it investigated how teacher characteristics and context influence the utility of recommendations. As such, the perspectives required for DPL could also affect why recommendations are considered to be useful, which we also explore in this study.

## 3 Methods

### 3.1 Educational recommender system

This study was conducted in the context of [project name], a learning portal for K12 education in the Flemish region of Belgium that offers lessons with learning activities from several educational applications [30]. Learning activities could be exercises or games in personalized learning environments or videos, audio files, and websites. An example of a learning activity is an exercise or an educational game on reading. Teachers on [project] can assign, monitor progress, and give feedback to students on a series of learning activities. This series is called a learning track. Learning tracks can have adaptivity, with branching paths based on cognitive, metacognitive, or affective criteria established by the teacher. Learning tracks on [project] could be made by teachers or pedagogical experts and vary in subject. For each learning track, teachers have the choice to share learning tracks or copy tracks from other teachers and experts. They could also adapt learning tracks to suit their needs, choosing learning activities from the library of [project]. Student actions on [project] consist of starting learning tracks, completing learning activities in learning tracks, and asking questions if there is a problem. During the evaluation of the pilot phase of the project, teachers expressed interest in using materials from other teachers. While expressing this interest, teachers also desired to easily find materials, leading to the development of an ERS for learning designs and materials for [project].

The developed ERS recommends items to teachers based on the activities of their class. The items in our system included both learning tracks and learning activities. We used student activations of learning activities and tracks of all students using [project name] as input for the recommender system. These activations were from diverse contexts and learning goals. In addition, we included side information, such as school as a student feature, activity type (not considered for tracks), duration (not considered for activities), grade level, and subject as item features to improve the recommender system. With data and side information from these diverse contexts, the recommender system is designed to capture all learning goals connected to their respective contexts. To justify learning track activations as input, students can be assigned multiple learning tracks and choose to complete a certain number of tracks. Therefore, student activations of learning tracks hold implicit information on a student's interests or motivation. To justify learning activity activations as input, we first noted that each learning track can branch out to different activities. Branches are selected based on the student's actions. For example, students completing a learning track with cognitive branches are moved between branches based on their performance on quizzes designed by the teacher. As such, student activations of learning activities hold implicit information on the performance of students and students' beliefs in their own learning or interests depending on whether the track has cognitive, metacognitive, or affective personalization. In this way, recommendations are more adaptive to how students perform on these personalized tracks.

School information was included as a categorical user feature stored in a matrix filled mainly with zeros (a sparse matrix). This was because one-hot encoding was employed to encode school information. One-hot encoding is a technique that creates columns for all options within a category, with the correct option marked as one and the rest as zeros. For item features, activity type and duration were also categorical features stored in sparse matrices. The activity types considered were exercise, video, website, image, and audio. Duration could be considered to be less than 1 h, one lesson, one to two lessons, or more than two lessons. The grade level and subject were categorical features stored as multisparse matrices, which means that the grade level and subject could be subdivided and stored in multiple matrices. All sparse matrices were connected to a layer of neural network specifically designed to transform sparse matrices into low-dimensional embeddings or numbers representing their meaning before connecting outputs from these layers into the recommender system model [31,32]. This process of transforming sparse matrices is learned during the training of the recommender system. An example of an observation in our dataset is presented in Table 1. Table 2 holds the size of the dataset used to train the recommender system and the size of the features.

**Table 1:** Example of a possible observation of the dataset, including user and item features.

User ID	Item ID	School ID	Activity type	Grade level of activity	Secondary school subject	Primary school subject
5	2	6	Exercise	Primary|second-grade		Natural science

**Table 2:** Dataset information.

Feature	Size
Students	8556
Teachers	637
Schools	218
Activities	4310
Activity type	5
Learning tracks	1484
Duration	4
Grade levels	55
Primary school subject options	131
Secondary school subject options	100

**Fig. 1:**
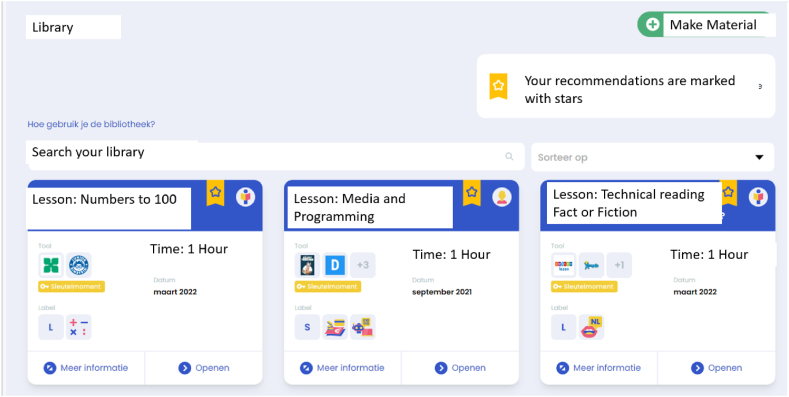
Teacher's view of the recommender system.

The recommender system uses a deep learning-based approach, the Deep Interest Networkdeep interest network architecture (DIN [33]). The system recommends items based on previous student activations of items as well as information about the student and item, with more weight given to more recent interactions. This model uses a mechanism similar to attention mechanisms to calculate the influence of item and student information on the likelihood of future usage. Importantly, the subject and grade levels of items as assigned by teachers are used as side information to generate recommendations. The influence of these interactions is learned from the student interaction data. This model was chosen owing to its success in offline tests in an earlier [project] pilot phase [34]. The DIN produces the probability with which a student will activate that learning track or activity based on previous tracks or activities. A maximum of 50 activities or tracks with the highest probabilities of use are generated. These items are then aggregated for a class by averaging the likelihood of use for all students as a form of score aggregation. Score aggregation was chosen due to research recommendations of greater efficiency and flexibility [35,36]. Items that are only recommended to one student are removed. Then, a maximum of 50 recommendations are displayed as recommended items to teachers when they go to their library for a learning track or when teachers create or adapt a learning track for learning activities. An example of a teacher's view when using the recommender system is presented in Fig. 1.

### 3.2 Evaluation design

To test the recommender system, we first recruited teachers who were using [project name]. After recruitment, the teachers were introduced to the recommender system. Next, they completed a think-aloud procedure using the recommender system. Subsequently, the teachers were tasked with creating lessons with and without the recommender system. After creating lessons, the teachers completed a semistructured interview based on the ResQue framework used by Fazeli et al. [7] and a survey using the Technology Acceptance Model (TAM) [37]. Finally, the results were analyzed using Interpretive Phenomenological Analysis (IPA), and the survey results were averaged to understand user satisfaction for the entire sample.

#### 3.2.1 Teacher recruitment

Six teachers were recruited for this study. They were teachers actively using [project] with an interest in personalized learning. The selection criteria were teachers that are active users of [project], teach at a K12 school with any subject, and have an interest in using personalized learning in their class. To be an active user, teachers must have copied at least one learning track for their own use or have assigned at least one learning track to their students, both within the previous 4 months. To express an interest in personalized learning, teachers must have signed up for a class on personalized learning given by the [project] team or previously contacted the [project] to ask for support or provide feedback. The teachers chosen for this evaluation were diverse, with a wide range of subjects and years of experience, providing us a broad perspective on the use of the recommender system. The secondary school teachers taught only a single subject (one taught English and the other taught Dutch), whereas the primary school teachers taught various subjects. The teachers also described class sizes that varied on the level and needs of the students. Table 3 presents the teachers’ experiences and contexts. After obtaining consent, the teachers were introduced to the recommender system and instructed how to use it to build or adapt lessons. Then, they were asked to create a small lesson for their class using the recommender system in a think-aloud procedure.

**Table 3:** Teacher characteristics.

Teacher	Years of teaching	Self-reported experience with TEL	Self-reported experience with personalized learning	Level taught
1	18	High	Very high	Secondary
2	3	Medium	High	Primary
3	8	Low	High	Primary
4	20	Medium	High	Primary
5	30	Low	High	Primary
6	19	Medium	Medium	Secondary

#### 3.2.2 Think-aloud procedure

In this think-aloud procedure, teachers were tasked with creating a small lesson with two to three activities, with prompts to think aloud about the recommender system, how they use the recommender system for personalized learning, if recommendations are useful, how they personalize in the classroom, and, finally, if the recommender system is helpful for personalization. This think-aloud procedure was adopted to answer the first research question: How do teachers in our study use the ERS in their practice? We answered this question by comparing similarities and differences in usage and comments on usage between teachers during the think-aloud procedure.

#### 3.2.3 Lesson choice

After the think-aloud procedure, the teachers were asked to create or adapt three lessons on the subjects of their choice at their own pace. Three lessons were selected so teachers could more easily compare and disentangle the effects of the recommender system from the effects of personalized learning while not being overburdened with workload. The first lesson was to be created using the tenets of personalization according to the teacher and without the recommender system. The first lesson was chosen to provide a baseline with which teachers could compare their experiences. The next two lessons were to be created with the recommender system and could be completed in any order. In this phase, one lesson was to be personalized and one lesson was to be created without personalization. Aside from these instructions, no other recommendations or requirements were given to the teachers. As such, the manner and target of personalization were decided by the teacher. For example, a teacher could choose to use one branching learning track that branches on cognitive ability a lesson, whereas another teacher could use multiple learning tracks based on affective interest for a lesson. These characteristics could have also been influenced by the topic decided by the teacher. Having the teachers develop the personalized and non-personalized lessons using the recommender system was intended for them to more clearly differentiate and compare experiences with the recommender system with and without personalized learning. In addition, the teachers were given the choices of subject, manner, and method of personalization to allow them to more accurately use the recommender system in their typical contexts.

#### 3.2.4 Interview and survey

After the creation, the teachers completed a semistructured interview and surveys on their experiences with the recommender system and personalized learning. These interviews and surveys were aimed to answer the second question, i.e., what are teachers’ perceptions toward the ERS used. Here, we used the ResQue framework previously utilized by Fazeli et al. [7] to assess another ERS and the TAM, a model used for evaluating other TEL applications measuring perceived usefulness, perceived ease of use, and intention to use to model usage behavior [37,38]. Both were selected as they were used with K12 teachers in previous research investigating recommender systems [7,39]. The recommender system was evaluated using quantitative and qualitative measures. More specifically, the elements of the ResQue framework were qualitatively evaluated and comprised perceived accuracy (or how accurate users think the recommendations are), novelty of recommendations (how new the recommendations are to users), diversity of recommendations (how diverse the recommendations are), serendipity of recommendations (how surprising or unexpected the recommendations are), and utility (how useful the recommendations are). To assess these criteria, we adapted the survey questions used in previous research to be more conducive for an interview by making questions more open-ended [6]. Utility was also prompted during the think-aloud procedure, with teachers being prompted to speak about how useful the system is to adapt and create classes while using the recommender system. We also qualitatively investigated recommendation quality (how good the recommendations are according to users) and teacher satisfaction. Recommendation quality was measured by asking teachers to rate the quality of their recommendations on a scale of 1–5 and to discuss the quality. Quality, satisfaction, and utility were also quantitatively measured. Satisfaction and utility were measured using a survey based on the TAM (Venkatesh & Davis, 2000). The quantitative measure of quality was also mentioned above.

These criteria and procedures have been developed from a review of literature investigating the evaluation of recommender systems for technology-enhanced learning by Ref. [28]. The interview questions and think-aloud procedures were initially transcribed. After transcription, the first author organized the teacher's comments into the evaluative criteria originating from the ResQue framework [7]. Within each of these evaluating criteria, individual teacher experiences, teacher comments, and teacher actions during the think-aloud procedure were compared for commonalities and differences. Then, common comments were compared between criteria to find similar experiences across the criteria. These procedures followed an IPA approach where the focus was on the description and comparison of the experiences of all participants within their own words [40]. The quality ratings of the recommendations were averaged to broadly describe the quality of recommendations for the group and were included in the IPA analyses. The TAM was scored on a 7-point Likert scale and used to describe satisfaction (using the intention to use subscale) and utility (using the perceived ease of use and perceived usefulness subscales) quantitatively.

Finally, the third research question was answered by deriving themes on DPL from the interview questions on utility previously mentioned and from other questions on how the recommender system impacts DPL. For example, “Is it now easier to find and include adaptivity in your teaching? Why (not)?” and “Do you believe that this recommender system has changed how you implement personalized learning?” were asked to teachers to gain an insight into how this recommender system impacted their work. We again analyzed these interview questions using an IPA approach, following the same procedure described above. To understand whether these effects described by teachers were local, during the open-ended interview process, we also asked if their opinions were specific to the presented recommender system or more general.

## 4 Results

In this section, we discuss the experiences and themes produced by our analyses. We answer our first research question on exploring teachers' use of the ERS by analyzing our think-aloud procedure. We then answer our second question on understanding whether the ResQue framework captures teachers' views. Here, we organize our results by the evaluation criteria from the ResQue framework. Within these criteria, we derive themes and connections from the interview and think-aloud analyses with additional context derived from the survey results. Finally, we answer our third research question on the exploration of teachers’ view of how the ERS and DPL interact by analyzing interview responses on questions related to this topic.

### 4.1 Teacher use of the teacher-centric educational recommender system

From the think-aloud task, the teachers differed in how they built lessons with the recommender system, with some building their own lessons with recommendations for activities and others adapting suggested lessons. The majority of teachers first decided the subject of the lesson. From there, some teachers investigated learning track recommendations. Teachers 4 and 5 wanted to see if there were tracks that they could use to decrease their workload. Each teacher found some options but instead decided to build their own lessons as they found the learning tools in those tracks to be more useful than the lessons themselves. During the building or editing of lessons, Teachers 1, 2, 3, and 6 started by focusing on the development or implementation of adaptive elements for their lessons. Here, there is a suggestion for future use from Teacher 6: “[In the future] will the recommender system be able to put something, er, come up with suggestions … ?” The teachers tended to prioritize adaptivity from the results of cognitive tests. Next, the teachers added activities. Most teachers initially judged the presented recommendations in terms of subject and difficulty level. Multiple teachers found some useful items but often considered items to be too difficult. This judgment differed in varying sections of the learning track as teachers built learning tracks envisioning prototypical students for adaptivity, with Teacher 3 commenting “This activity is too hard for [these students] … but it might be useful later [for the advanced students] …” from Teacher 3. However, difficult items could act as inspiration, with Teacher 4 finding activities in the same tool as the difficult activity.

### 4.2 User-centric evaluation of the educational recommender system

#### 4.2.1 Accuracy and quality

First, the ratings of the recommendations were middling, with an average of 2.9 (*SD* = 0.9) on the 5-point rating scale. This moderate average was related to various issues brought up during the interview. One issue with accuracy was that the recommender system at times recommended teachers with a different version of their own content. Teacher 1 supposed that this was due to the amount of content for their subject: “There is a lot of content for math or science, but not for language.”

The second problem was that the teachers believed that recommendations were at the wrong difficulty level. According to Teacher 1, “The recommendations were accurate as in the topics were mostly accurate, but the level often wasn't.” This sentiment is echoed throughout the interviews and think-aloud procedures. In the think-aloud procedures, this sentiment was observed in both actions and comments. The teachers repeatedly selected a recommended item, explored its contents, and then clicked away. Teachers 2 and 3 supposed that for them, this incorrect level is due to the diversity found in their classrooms along with the lower level of their students. Teacher 2 stated: “I work with children from [a low level] to [a medium level]. … I would like to choose the level and subject of the activities [that I am recommended].” Student diversity also had an influence on Teacher 6, but they thought that accuracy in subject is far more important than accuracy in level: “… because level is always very difficult to estimate because maybe the level of my students that are in the second year of English is worse than somebody else's students who are in the second year of English.”

#### 4.2.2 Novelty, diversity, and serendipity

The evaluation of novelty, diversity, and serendipity varied between teachers. However, a commonality was that the recommendations that fit these criteria were perceived to be unhelpful at best and stressful at worst. Teacher 4 described a scenario in which the recommendations were novel, diverse, and surprising, causing stress: “… the theme was Belgium, and I went to search, and there was the king, and the provinces, and the neighboring countries, and this too, and I thought, I just want to make one learning track … there was naturally too much.” Such scenarios and considerations were stressful for Teacher 5: “[having too many options and subjects] I am always afraid that I am missing something. That's not good work, that's not good.” Other teachers noted that it is unnecessary to have novelty, diversity, and serendipity in recommendations, as teachers have to follow a curriculum and other plans. As Teacher 3 stated, “Your lesson that you must give, there is little room for other things ….”

For novelty, Teacher 1 found very little novelty in their recommendations but connected this lack of novelty to the lack of content for their particular subject. Multiple teachers found that the recommender system suggested some novel items, but the usefulness of those items questionable. Valuable novelty came from new tools or using new learning tracks as a base for other lessons. Teacher 4 found value in the introduction of new tools to use in their classroom: “I found things there that when I went back to the apps that had value, because I couldn't use the learning track or the learning activities in my class … I would filter for my level and search in the app, and then find simple activities to use.” However, Teacher 6 perceived this as negative as they did not want to “plow through every learning tool” to find activities that would fit their classes. Similar to the novelty of new learning tools, Teacher 5 found learning tracks with ideas that they wanted to teach but were at the wrong level, so they used that learning track as a base: “There was one learning track on continents … But there were two activities that I could not open, so I changed them and used it with my colleague. But, this isn't the time saving that we were hoping for. It is time-consuming to make something new myself.”

In general, for diversity, some teachers believed that there was too much diversity in recommendations, whereas others thought that the amount of diversity was just right. Teacher 6 summarized the general desire for diversity for the recommender system as “I want 1 topic, with 3 levels. 20 items is already too much.” They made the specific distinction of stating that items should be able to fit on one screen to not be too much. Similarly, the best serendipitous items were in types of activities, according to Teacher 6. However, the majority of teachers believed that serendipity was not useful for their work.

#### 4.2.3 Satisfaction

From the TAM intention to use subscale, teacher satisfaction was good, with an average score of 5.1 (*SD* = 1.2) on a 7-point rating scale. However, teachers' satisfaction during the interviews differed. Teachers 1, 2, 3, and 6 were not satisfied for different reasons but all stemmed from the same issue: the current version of the recommender system is not as helpful as desired. For Teacher 1, this stemmed from the aforementioned difficulties with content and not from the recommender system. Teacher 6 agreed with difficulties not directly from the recommender system, but not content, rather due to the necessity for them to go through new learning tools. Contrarily, Teachers 2 and 3 attributed their difficulties with the recommender system to the high amounts of novel and diverse items presented to them. Teacher 2 stated: “The recommender system wasn't helpful for me because I found it easier to … search and use the filters. That was more [immediately] helpful.” Teachers 4 and 5 were satisfied with the recommender system but did not disagree with any of the opinions from previous teachers. Rather, they viewed it as a promising first step. As Teacher 5 stated, “I liked it as an experiment, but I hope it will come out more focused. I really, really want this to continue.”

#### 4.2.4 Utility

Utility as measured by the TAM was positive, with an average score of 5.1 (*SD* = 1.8) on the perceived ease of use subscale and an average score of 4.6 (*SD* = 1.2) on the perceived usefulness subscale, both measured on a 7-point Likert scale. However, the utility in the interviews was more varied. The aspects of utility in the interviews are split between two concepts, opinions on the simplicity and the actual usability of the recommender system and the broader concept of how the recommender system impacts their teaching practice. For the first concept, teachers generally found the recommender system easy to use. Teacher 1 specifies what aspects contribute to this ease of use: “The visualizations are very clear, …it's just, err, it nicely integrates with the platform itself.” Only Teacher 6 had issues with the simplicity of use, “I can see that I have a recommender system, but I can't see them in my search engine screen,” describing that when she tried to search for items, recommendations disappeared.

Despite the relative ease of use, the teachers felt that the recommender system was not particularly useful to their teaching practice. They evaluated the utility in regards to time-saving during lesson preparation. Teacher 6, in particular, was most explicit about this evaluation of utility, stating “[the best recommendations] are useful, so if they shorten your time of preparation ….” This is in line with usage during the think-aloud procedure, where one teacher suggested further time-saving utility to be added in the future. However, some teachers found recommendations as inspiration useful. As Teacher 5 stated, “I do find [using the recommendations as inspiration] good. Yeah, it sees things that I go, oh yeah, I've taught this, I could use it for practice, so I don't have to teach that again.” This is seen in practice during the think-aloud procedure, where Teacher 3 suggested that a recommendation may be useful for advanced students and Teacher 4 found inspiration within a suggested activity.

The teachers also commented on future use, both for themselves and for other teachers. The majority of teachers felt like they would not use the recommender system in its current state; however, two differing themes for use emerged: utility for less skilled teachers and improvements for future use. Teacher 1, in particular, clearly claimed a utility for less skilled teachers: “[when less skilled teachers use project] I think the recommendation system will be more useful then than it is now. … I think it could be the first step in using things like [project], where you take something that is already made, and you adjust it a little bit and then you use it and, um, I think that could be the first step [into personalized learning] ….” This is also seen in how other teachers commented on their use. Contrarily, Teacher 6 mentioned that their colleagues wanted to use the recommender system as an interactive tutoring system: “I can speak for my colleagues for that because they thought that [project] would be able to do that, umm, that the recommender system can say … okay this is what you need, you have to practice more on this, these are the exercises, do this. … and then you can walk around like a coach.” However, the teachers in this sample disagreed, wanting more control over which recommendations are seen. Many teachers suggested filters before recommendations, specifically filter first on level and then on subject. Teacher 3 explained: “Because you, as a teacher, work on language, maths, and in the end, all of your recommendations get mixed up. Then, with the filters, you can say, oh, I want to work on maths now.” Despite the general desire of filters, the specificity of those filters is debated. Teacher 4 wanted very specific filters, stating “I want very, extremely specific filters, starting with the subject, then specific topics within that subject.” However, Teacher 2 disagreed, stating “… it would be too difficult if you made everything too specific.” They noted that this difficulty is both for the teacher and the system, as some very specific topics can be taught in multiple levels with the same material. There is also the feeling that too specific filters would remove too much agency from the teacher, with Teacher 3 glibly stating “I want to work a bit too, eh?”

### 4.3 Teachers’ views of the educational recommender system and digital personalized learning

The teachers believed that the recommender system did not affect their current workflows or pedagogical decisions; however, they all mentioned the potential for major benefits of the recommender system for DPL. They also mentioned that a recommender system can serve as a first step in DPL. Most explicitly, Teacher 1 commented on this potential and Teacher 5 demonstrated aspects of that potential. However, teachers more generally believed that recommender systems can affect better DPL by saving teacher's time. As Teacher 3 summarized, “I think [the recommender system] would save a lot of time. If you can say, …okay I can use this learning track for these students, and you spend less time searching and going step by step checking everything. … [even using the learning track as a base], you don't always need to think about what you need to start or make for other [students].” Time saving can also lead to easier teaching and DPL, according to Teacher 4: “You can differentiate even more. The more time and proposals the teacher has, the better they can respond to the need of each child.” Teacher 6 makes a point in specifying the production of diverse recommendations in level for bettering DPL. However, Teacher 1 supposed that these benefits were moderated by the amount of content within the system itself.

## 5 Discussion

In summary, we investigated a recommender system for teachers in K12 education, its recommendations, and its impact on the delivery of DPL. This study focused on the utilization of the opinions of experts to provide a better understanding of how teachers can use recommender systems and if a specific user-centric evaluation captures our teachers’ values during the recommender system evaluation. In RQ1, we were specifically interested in exploring how teachers use the recommender system in their practice. We found that when using the recommender system for lesson development, the teachers in our study preferred using the recommender system to build new lessons rather than adapt new lessons. In RQ2, we investigated how teachers in our study evaluated the recommendations presented and recommendations as a whole using a user-centric evaluation framework as a base during teacher interviews. The teachers in this study evaluated recommendations on their ability to save time. For recommendations to save time, the interviewed teachers believed that recommendations should be accurate to subject and level, with the additional option to first select which subjects and levels recommendations are seen. Finally, in RQ3, we asked teachers about the impact of the recommender system on their delivery of DPL. The teachers stated that the recommender system in its current state does not have an impact on DPL. However, they saw tremendous potential in recommender systems to affect better personalization by easing teacher burdens during the preparation of DPL. Notably, based on this small sample, we cannot claim that our results are generalizable or representative for the views and opinions of the population of teachers, but we still believe our study provides insights that are transferable, in the sense that they can inspire the development and implementation of future ERSs. Below, we discuss the connections to previous research and reflect on how this research can relate to the implementation of DPL and aid the future development of recommender systems.

### 5.1 Exploring recommender system use

Previous investigations into teacher-centric recommender systems have demonstrated that researchers typically aim to accomplish two goals: improve teaching practices and recommend quality content [19]. However, in the K12 context, the focus for teachers is to recommend content that can quickly be integrated into a lesson on a particular subject. From the think-aloud procedures and interviews in our study, there is a common experience, which we summarize below.

First, the teachers in our study had a topic or subject that was the focus during a session where they used the recommender system. We believe that for our teachers' experience, this decision is important and challenging. To select this topic, the teachers in our study synthesized information about curricula, their class's point in a curricula, the skill levels within their class, and various other information based on their previous experiences, attitudes, and pedagogies to select what topic to prioritize during the use of a recommender system. Curricula are particularly important as. As Teacher 3 stated, “Your lesson that you must give, there is little room for other things ….” As such, the choice of topic may be a difficult decision depending on the teacher's skill, experience, attitudes, and particular needs. Following the comment of Teacher 4, “I want very, extremely specific filters …,” teachers would prefer to first select the subject and possibly level before viewing recommendations. This filtering approach proposal and subsequent reduction of options may be an attempt to reduce the cognitive load of choosing activities caused by information overload or fears of missing the required materials. This can be seen in the comment of Teacher 5: “[having too many options and subjects] I am always afraid that I am missing something.” Next, the teachers interpreted recommendations by estimating the congruency between the recommended items' topic and topic selected during the selection stage as well as the congruency between the items' level and the level of the envisioned archetypical student or groups of students. Finally, the teachers used recommendations to create lessons for their classrooms.

In addition to how teachers use recommender systems, more recent research has investigated how teachers integrate and collaborate with educational technologies such as recommender systems in their classrooms. Lin et al. [21] proposed that recommender systems aid in before-class preparation by generating recommendations mainly from student actions. While our research agrees with the positioning of recommender systems, the teachers in our study also felt that recommender systems should generate recommendations considering broader contextual features such as the previously mentioned curricula. These broader contextual features have a great impact on teacher decisions, as implied by Teacher 3. In the model of Lin et al. [23], the subject is included as side information. Contrarily, Teachers 3, 4, and 6 believed that more specific information should be used in the recommender system. We believe that including the structured nature of curricula and learning goals may provide more accurate, useful recommendations in the K12 context, as evidenced by Teacher 3's statements that curriculum considerations limit the choices that a teacher can make,. In addition, providing teachers with the explicit choice of selecting which learning goals to work on may provide more usability, as suggested by the teachers in this study. Aside from the explanations provided by the teachers themselves, other research on educational technology noted that teachers are often educational designers [41]. We inferred that the implementation of these recommendations may result in the development of a recommender system that better reflects their work as educational designers. In addition, other researchers have proposed models that specify interactions between teachers and educational technology, such as the model of Molenaar [17]. The teachers in our study suppose that teacher-centric recommender systems should be closer to teacher control than full automation, with recommender systems aiding in reducing workload. However, its placement within the model is debated, with some teachers preferring aids with mainly human control, seen in Teacher 3's comment, “I want to work a bit too, eh?,” whereas others view it as a form of conditional automation, as seen in Teacher 6's comment of “okay this is what you need, you have to practice more on this, these are the exercises, do this. … and then you can walk around like a coach.”

In previous research, investigations of user experience can improve user satisfaction [42]. Previous research theorizes that improvements in user satisfaction come from increasing the knowledge about a user's context, enabling designers to better consider different algorithms and designs to help users [43]. In practical terms, other research supposed that integrating data about a user's experience (found in user experience studies) into the algorithms driving recommender systems further increases user satisfaction [42]. In the perspective of education, this study agrees with previous research demonstrating that conducting user experience studies with teachers and integrating or considering teachers' experiences when designing recommender systems can increase the usage of teacher-centric ERSs [39]. Therefore, for future work, researchers should investigate how different levels of control can be supported for teachers. While such research has been conducted in other contexts, little work has focused on the different control levels for ERSs [44]. More work is also needed on how the presentation of a recommender system contributes to cognitive load in teaching settings, particularly K12.

### 5.2 Evaluating recommender systems

Previous ERSs have been mainly evaluated on recommendation accuracy, user satisfaction, and pedagogical effectiveness [6]. However, for teachers, these metrics are either difficult to capture (in the case of pedagogical effectiveness) or fail to completely assess the quality of a recommender system. In response, user-centric evaluations have been conducted to gain better understanding of user satisfaction [7]. Similar concerns have led to the rise of human-centered learning analytics, a field that focuses on the development of applications based on learning analytics, such as recommender systems, that include stakeholders in the development and evaluation [45].

However, our results indicated that in this study, Fazeli et al.’s [7] ResQue framework did not seem to match the teachers' views and opinions on what is important for recommendations in the K12 context. This can be seen in Teacher 5's stress on the inclusion of novel and diverse recommendations or Teacher 6's general statement against diversity: “I want 1 topic, with 3 levels. 20 items is already too much.” Beyond the quality framework, we found disagreement between the TAM results and the impressions noted in the interviews. In particular, some teachers believed that diversity and serendipity were not important and should not be considered during evaluation. The potential of recommendations based on these two qualities to increase workload and stress was brought up by teachers, as seen in the above comments. These two tenets of quality are also not indicative of a teacher's responsibilities, as implied by Teacher 3 in their comments on curricula. As mentioned earlier, according to our sample, a recommender system for teachers is not a tool for exploration or discovery. The focused nature of recommender systems is likely not dependent on pedagogical strategy, and capturing this focused nature should be a priority during evaluations in the K12 context. These results match a previous review on ERSs stating that evaluating user satisfaction alone is insufficient in judging the educational support offered by ERSs [6].

Teachers focus on specific elements within the broader concepts of accuracy and utility for evaluation, which may explain the discrepancy between the TAM and interviews. Accuracy refers to how closely recommendations match the topic and level chosen for a specific lesson. This can be seen in Teacher 1's evaluation of recommendations: “The recommendations were accurate as in the topics were mostly accurate, but the level often wasn't.” Utility for teachers in our study is regarded as time-saving. Specifically, teachers in our study judge if the recommender system help develop lessons quickly. This is explicitly stated by Teacher 6: “[the best recommendations] are useful, so if they shorten your time of preparation ….” Here, teachers concretely envision the reduction of orchestration load by technology. Contrarily, the TAM evaluates if the system is generally useful and simple to use without defining what makes a system useful [37].

In other tests of teacher-centric recommender systems conducted on teachers, accuracy and utility have diverse conceptualizations and are often tested separately. In Ref. [2], the utility of the recommender system was measured as increased usage of the recommender system. However, this evaluation of utility did not come from teachers and was based on the use of social trust for recommendations. According to Ref. [7], accuracy does not relate to a teacher's user satisfaction. This may be due to a difference in conceptualization between the ResQue framework and how the teachers in our study conceptualized accuracy. In another study, accuracy was regarded as how closely the recommender system matches teacher decision-making processes [46]. This conceptualization of accuracy matches more closely with how teachers conceptualized accuracy in our study. Furthermore, in our study, conceptualizations of accuracy and utility were intertwined, with high accuracy leading to less work needed to select lessons.

When evaluating recommender systems, conceptualizing accuracy as how well recommendations adhere to expected lessons and utility as how well the ERS reduces workload could help evaluations of ERSs. Such evaluations can more accurately account for their users' experiences by relating more closely to the educational realities of users. Furthermore, research on other teacher-centric applications demonstrated that focusing on these aspects can lead to the development of more effective ERSs. Ley et al. [47] reviewed other model-based teacher-centric applications and proposed that the focus of these applications on mimicking the logic and pedagogical reasoning of teachers leads to deeper and more useful support. This could be attributed to the fact that teachers can better understand the pedagogical decision-making involved, resulting in reduced workload. Moreover, Ley et al. [47] proposed that applications that mimic teacher decision-making can help teacher professional development, as providing actions or recommendations to improve student learning can show teachers what can be done for student learning. The teachers in our study agreed with this point, and this is discussed further below. These aspects of accuracy and utility can be integrated into a new questionnaire for evaluation of recommender systems for teachers, as questionnaires are often used to conduct user studies [6]. However, more qualitative studies on teachers using recommender systems should be conducted, particularly in different contexts and with different pedagogical strategies, as this research may not fully generalize to other contexts. Finally, in the future, recommender systems could be built to reduce the difference in students’ previous activities and recommended activities by only considering recommendations that match in subject and the curricula of the region as positive feedback to target for the recommendation algorithm.

### 5.3 Effects on digital personalized learning

In terms of DPL, the teachers in our study believe that the recommender system has two functions: initial onboarding for less skilled colleagues and as an orchestration tool for reducing workload. For the first function, teachers envision other teachers starting with DPL to initially explore lessons, change some to fit their context, and then, as they become experts, create their own lessons more tailored to their students and context. As explicitly stated by Teacher 1, “… I think it could be the first step in using things like [project], where you take something that is already made, and you adjust it a little bit and then you use it and, um, I think that could be the first step ….” We suppose that this “first step” reduces the need for technological knowledge, enabling the teachers to start with new technology and personalized learning. This is corroborated by the results of previous research indicating that these technical supports are extremely important to teachers and could motivate teachers who do not use technology or DPL to include such tools and pedagogies into their teaching [48]. The connection between technology skills and personalized learning is well established in the literature, as teachers considered the most experienced and effective with personalized learning also have high skills with technology [13,49,50]. Moreover, even though the need for technological knowledge is reduced, further training on digital skills may still be helpful. Here, in combination with teaching teachers how to use technology in their classroom, teacher development programs can teach them how to better interact with recommender systems. This can mean training them to use filters more actively or identify good materials from other teachers. Teacher training can also train teachers to alter recommended learning designs to further personalize learning, as this was rarely done in our study. Beyond training teachers to use recommender systems, recommender systems can also serve as professional development to train teachers. The “first step” described by Teacher 1 fits the theme of providing support during instructional design that is important for the delivery of teacher professional development (both on the job and in formal teacher education) for digital skills [51]. In our case, providing recommendations when altering or developing new learning designs can support new teachers as they develop and experiment with instructional design. In addition, we suppose that after viewing and using such premade learning designs, teachers can improve their skills by modeling their learning designs from the experiences and examples recommended by the recommender system. As such, recommender systems purely as a technical aid can help with the proliferation and quality of DPL in K12 education and aid in teacher professional development.

For the second function, the teachers in this study believe that a recommender system has the main purpose of reducing the workload of teachers. An example is Teacher 4 who believes that with a recommender system, “You can differentiate even more. The more time and proposals the teacher has, the better they can respond to the need of each child.” However, it is unclear whether this is specific to ERSs. In a case study in a school that first implemented personalized learning, Bingham (2017) found that despite the wide variety of tools and supports available for teachers, they were all perceived as an attempt to reduce teacher workload. Therefore, the opinions of our teachers may just reflect their opinions on the role of educational technology in the classroom. Even so, the teachers in our study believe that the reduction in workload leads to more effective DPL by allowing the teacher to focus more on diversification for specific groups of students within their classroom. However, previous research demonstrated that for these tools to be most effective, they should allow for freedom of choice in finding other materials or other tools [52]. In other words, tools such as recommender systems should not limit a teacher's autonomy. However, how much automation limits autonomy remains unclear. Some teachers believe that recommender systems should function similar to an intelligent tutoring system, where the teacher makes an initial choice of subject and then the recommender system recommends activities to students for them to perform automatically. Other teachers, such as Teachers 2 and 4, believe that they should be responsible for selecting activities and even be involved in generating recommendations. However, even within these teachers, there is disagreement. In this study, teachers disagree on how much influence they should have, with some only wanting to select very basic features to see recommendations, such as Teacher 2, and others wanting specific, granular control over the subjects and levels of recommended items, such as Teacher 4.

Future research should also investigate at what point is there too much automation with recommender systems. Future research could also explore how teachers new to personalized learning or preservice teachers initially use recommender systems to explore the pedagogy. However, more generally, researchers should determine what teacher characteristics influence recommender system use, similar to current research lines for dashboards [27]. For personalized learning, future research should investigate whether recommender systems produce direct effects on student's learning performance.

### 5.4 Limitations

This research is not without limitations. First, teachers had limited time to accomplish their tasks, adding time pressure that may have reduced the quality of recommendations, as the recommendation algorithm may not have had sufficient data to quickly respond to changing teaching focuses. This time pressure may have also reduced the quality of teacher use. Also related to time, some teachers had only used [project] for a short period of time before using the recommender system, leading to the possibility of cold start effects in our research, as the recommender system may not have had sufficient student data to accurately model the teacher preferences of new teachers. Another possible limitation was the amount of content. As noted by teachers, certain subjects or topics had few learning activities or learning tracks that could be recommended, leading to poor accuracy for some teachers. As such, with more content in [project], teacher ratings of accuracy and satisfaction may have improved but likely would not have changed any patterns of use or opinions on evaluation. In relation to the limited amount of content, we were unable to disentangle the effects of the subject chosen and therefore the content and effects of the recommender system. As such, perceptions about our recommender system could be affected by the subjects and topics chosen by the teacher, as some teachers commented. More broadly, our sample was small and experienced with personalized learning, making it difficult to generalize the results to other teachers. These teachers who were experienced with personalized learning may also have had a set pattern of working and more perceived control over their classroom, thus biasing them against further automation of education or, more broadly, perceived loss of control over their classrooms with additional technology. Moreover, the teachers in our study may have given socially desirable answers rather than report their true feelings. Therefore, our results may not completely reflect teacher opinions on recommender systems. Finally, only one teacher-centric ERS was studied in a specific K12 context. Therefore, the findings may not generalize to other types or implementations of teacher-centric ERSs or other contexts. As such, our proposed models and conclusions need further validation in different contexts.

## 6 Conclusion

This study evaluated a K12 ERS for teachers and aimed to understand how teachers use recommender systems in their practice and how these systems affect DPL. In general, the teachers in our study used the recommender system to create rather than adapt lessons. The teachers also shared multiple commonalities when using the recommender system, which we describe as a shared path. In terms of evaluation, specific conceptualizations of accuracy and utility of ERSs were of utmost importance to teachers in this study and could be integrated into user-centric frameworks for teacher recommender system evaluation to better match the views of the teachers in our study. Finally, according to the comments of the teachers in our study, recommender systems can aid in DPL by serving as a first step for effective differentiation and reduce teacher workload and stress during the preparation of personalized lessons. Notably, these effects are theoretical, as the recommender system in this study did not affect personalized learning. However, these effects match previous research on other technological aids for personalized learning. The teachers in our study believe that with decreased workloads, they can spend more time on addressing the needs of the students. To account for the conceptualizations of accuracy and utility stated by the teachers in our study, future recommender system evaluators should include the cohesion between teacher lesson plans and recommendations to evaluate accuracy as well as the amount of workload reduction to evaluate utility in future evaluations of teacher-centric recommender systems. Researchers interested in personalized learning can determine whether the addition of a recommender system has any direct effects on student learning and whether recommender systems make transitioning into personalized learning easier.

## CRediT authorship contribution statement

**Sohum M. Bhatt:** Writing – review & editing, Writing – original draft, Visualization, Methodology, Investigation, Formal analysis, Data curation, Conceptualization. **Katrien Verbert:** Writing – review & editing, Supervision, Methodology. **Wim Van Den Noortgate:** Writing – review & editing, Supervision, Methodology, Funding acquisition.

## Data Availability statement

No data is shared. The authors do not have permission to share data.

## Declarations

This research was given ethical approval by the Social and Societal Ethics Committee of KU Leuven (G-2022-5433-R3(AMD)).

## Funding

This work was supported in part by the Flanders Agency of Innovation & Entrepreneurship under grant number AH.2019.05.

## Declaration of competing interest

The authors declare that they have no known competing financial interests or personal relationships that could have appeared to influence the work reported in this paper.
